# The phosphodiesterase-4 and glycine transporter-1 inhibitors enhance in vivo hippocampal theta network connectivity and synaptic plasticity, whereas D-serine does not

**DOI:** 10.1038/s41398-020-00875-6

**Published:** 2020-06-18

**Authors:** A. Ahnaou, T. Broadbelt, R. Biermans, H. Huysmans, N. V. Manyakov, W. H. I. M. Drinkenburg

**Affiliations:** Department of Neuroscience, Janssen Research & Development, A Division of Janssen Pharmaceutica NV, Turnhoutseweg 30, B-2340 Beerse, Belgium

**Keywords:** Pharmacodynamics, Hippocampus

## Abstract

Dysfunctional N-methyl-D-aspartate receptors (NMDARs) and cyclic adenosine monophosphate (cAMP) have been associated with deficits in synaptic plasticity and cognition found in neurodegenerative and neuropsychiatric disorders such as Alzheimer’s disease (AD) and schizophrenia. Therapeutic approaches that indirectly enhance NMDAR function through increases in glycine and/or D-serine levels as well as inhibition of phosphodiesterases that reduces degradation of cAMP, are expected to enhance synaptic strength, connectivity and to potentially impact cognition processes. The present in vivo study investigated effects of subcutaneous administration of D-serine, the glycine transporter 1 (GlyT1) inhibitor SSR504734 and the PDE4 inhibitor rolipram, on network oscillations, connectivity and long-term potentiation (LTP) at the hippocampi circuits in Sprague-Dawley rats. In conscious animals, multichannel EEG recordings assessed network oscillations and connectivity at frontal and hippocampal CA1–CA3 circuits. Under urethane anaesthesia, field excitatory postsynaptic potentials (fEPSPs) were measured in the CA1 subfield of the hippocampus after high-frequency stimulation (HFS) of the Schaffer collateral-CA1 (SC) pathway. SSR504734 and rolipram significantly increased slow theta oscillations (4–6.5 Hz) at the CA1–CA3, slow gamma oscillations (30–50 Hz) in the frontal areas and enhanced coherence in the CA1–CA3 network, which were dissociated from motor behaviour. SSR504734 enhanced short-term potentiation (STP) and fEPSP responses were extended into LTP response, whereas the potentiation of EPSP slope was short-lived to STP with rolipram. Unlike glycine, increased levels of D-serine had no effect on network oscillations and limits the LTP induction and expression. The present data support a facilitating role of glycine and cAMP on network oscillations and synaptic efficacy at the CA3–CA1 circuit in rats, whereas raising endogenous D-serine levels had no such beneficial effects.

## Introduction

N-methyl-D-aspartate receptors (NMDARs) and cyclic adenosine monophosphate (cAMP) play a pivotal role in plastic mechanisms of learning and memory^1^. Dysfunctional NMDARs and cAMP signalling have been associated with deficits in synaptic plasticity and cognitive decline found in neuropsychiatric and neurodegenerative disorders such as schizophrenia and Alzheimer’s disease (AD)^2–4^. Therapeutic approaches that enhance NMDAR function through increases in endogenous ligands of the NMDAR, as well as inhibition of phosphodiesterases, which reduces degradation of cAMP, are expected to enhance endogenous neurorepair and synaptic strength to potentially impact cognition processes^5–7^.

The strength of the glutamatergic neurotransmission is tightly controlled by the synaptic concentration of glycine and D-serine near NMDA receptors. Glycine and D-serine are endogenous ligands at the glycine B site of the NMDA receptor, which act as a requisite co-agonist of glutamate for the activation of this receptor^8^. Glycine, which generally acts as an inhibitory neurotransmitter, has an excitatory activity at the strychnine-insensitive coagonist site^8^. D-serine, which is released from astrocytes is more potent at the strychnine-insensitive binding site than glycine^9^. On the one hand, levels of synaptic glycine are tightly controlled by the specific transporter GlyT1 localized on glial cells and neurons closely associated with the NMDA receptor^10^. Several well tolerated, high affinity GlyT1 inhibitors have been developed and shown to increase central glycine levels for a positive functional impact on central glutamatergic transmission and to possess the preclinical profile of putative antipsychotics properties in preclinical animal models^11–14^. On the other hand, reducing D-serine levels impairs NMDAR-mediated processes in several structures, including the hippocampus, prefrontal cortex, nucleus accumbens or amygdala. Functional studies using enzymatically, or genetically induced depletion of D-serine showed reduction of synaptic NMDARs currents and thereby alteration in synaptic plasticity at the level of the hippocampus^9,15,16^, amygdala^17^, and nucleus accumbens^18^, the retina^19^ and the hypothalamus^20^. The role of D-serine at NMDARs is further illustrated by studies showing that synaptic and cognitive impairments during aging is linked to a downregulation of D-serine synthesis^21,22^. Detailed analysis of the contribution of the two co-agonists in the regulation of NMDARs at the hippocampus CA1 level revealed that D-serine would preferentially act on synaptic NMDARs whilst glycine would modulate extra-synaptic NMDARs^15^.

The integrity of the hippocampal formation is critical for normal memory function, hence much experimental interest focused to characterize structural and functional changes of the hippocampus throughout aging and in disease animal models. Key mechanisms proposed to explain impaired cognitive processing are associated with deficits of network oscillations at the fronto-hippocampal circuit and impaired synaptic plasticity related to long-term potentiation (LTP)^23,24^.

Network oscillations represent fundamental mechanisms enabling coordinated activity between multiple association regions during normal brain functioning. Hippocampal theta oscillations have been found to drive processing in the prefrontal cortex^24,25^. Increased gamma band (30–100 Hz) oscillations occur during the transient brain states that are associated with attention and stimulus recognition^26,27^. More recently, several studies have suggested that gamma oscillations nested within theta (4–12 Hz) oscillations play a role in working memory functions^24^. Also, substantial data suggest that corticothalamic^28,29^ and hippocampal networks^30^ make use of beta (12–30 Hz) and gamma (30–100 Hz) frequency band activities for long-distance transmission of information among task-related brain sites, although a number of those studies were carried out in brain slices or animal model of diseases^31–33^.

LTP is most commonly induced by a combined activation of α-amino-3-hydroxy-5-methyl-4-isoxazolepropionic acid (AMPAR) and NMDA receptors. NMDAR-activity-dependent LTP is suggested as a mechanism for short- and long-term memory acquisition^34,35^. Presynaptic depolarisation leads to exocytosis of glutamate into the synaptic cleft, activates many of the postsynaptic proteins, including the cAMP. cAMP/PKA and cyclic guanosine monophosphate (cGMP)/protein kinase G (PKG) pathways involved in the LTP expression, maintenance and memory enhancement^36^. To regulate the signalling of both pathways, the phosphodiesterase (PDE) enzyme family hydrolyses cAMP and cGMP preventing kinase activity^3^. There are 11 PDE subgroups found in varying levels across the nervous system. There is a strong case for the regulation of synaptic plasticity by PDE4, which is the most widely studied PDE and is selective for cAMP over cGMP. PDE4 hydrolyses cAMP and is found in the hippocampus and cortex among other areas in rodents^37^. Rolipram exhibits memory-enhancing effects in rodents. A decrease of PDE4 isoforms has been shown in AD patients and the PDE4 inhibitor rolipram has demonstrated memory enhancements^36^ as well as displaying a good antidepressant effect but with unpleasant side effects^38^. PDE4 inhibition rescues impaired LTP and prevents object recognition memory deficits in an animal model of psychosis^39^.

In the present work, we modulated D-serine, glycine and cAMP levels to ascertain their functions in network oscillations and synaptic plasticity in healthy rats. By increasing synaptic concentration of glycine or D-serine in the vicinity of NMDA receptors, blockade of GlyT1 and/or D-serine are expected to potentiate glutamatergic transmission. We demonstrate that increased levels of glycine and cAMP increased hippocampal network activity and LTP in healthy rats. Under physiological conditions, synaptic plasticity in vivo at CA1 did not require high levels of D-Serine.

## Materials and methods

### Animals

All experimental procedures were conducted in strict accordance with the guidelines of the Association for Assessment and Accreditation of Laboratory Animal Care International (AAALAC), and with the European Communities Council Directive 2010/63/UE of 22/09/2010 and were approved by local ethical committee. Experiments were carried out on Sprague-Dawley male rats 180–250 g (Harlan Nederland) housed in individually ventilated cages located in a sound-attenuated chamber under the controlled light/dark cycle (light: 7.00–19.00) with standard food and water available ad libitum.

### Surgery and electrode implantation

#### Network oscillations and connectivity

Surgery was performed under Isoflurane anaesthesia as described earlier^40,41^. In brief, animals were equipped with two stainless steel fixing screws (diameter 1 mm) for the recording of frontal electroencephalographic activities (EEG) inserted bilaterally in the left and right cortex (FL/FR: AP + 2 mm, L ± 2 mm from Bregma). Four stainless steel wires used for intra-hippocampal electrodes (CA1: AP −3.14 mm, L ± 1.8 mm, V + 2.8 mm; CA3: AP −4.5 mm, L ± 3.8 mm, V + 4 mm from Bregma, respectively). In addition, stainless steel wires (7N51465T5TLT, 51/46 Teflon Bilaney, Germany) were placed in the muscle of the neck to record the electromyogram activity (EMG). Electrodes were connected to a pin (Future Electronics: 0672-2-15-15-30-27-10-0) with a small insert (track pins; Dataflex: TRP-1558-0000) and were fit into a 10-hole connector, after which the whole assembly was fixed with dental cement to the cranium. Animals were given at least 2 weeks as a recovery period.

### Recording, analysis of spectral oscillations and network connectivity

EEG recordings were derived from six brain regions under vigilance waking condition during the dark circadian phase^40,41^. Additionally, a general motion level was monitored in the home cage by two passive infrared (PIR) detectors placed above each recording cage, and artifact-free waking epochs with low-voltage fast EEG activity, high to moderate EMG and body activities were considered in the analysis. Epochs with high-voltage slow cortical waves in the absence of EMG and locomotor activities were discarded. A notch FIR filter at 50 Hz was applied to avoid voltage related to power line interferences.

Baseline EEG recordings of 30 min started 2 h after the light offset to avoid confounding circadian effect on EEG. Afterwards, EEG signals were recorded for 3 h after vehicle or drug administration (*n* = 8 animals for each condition). Continuous EEG and EMG field potentials were acquired at a sampling rate of 512 Hz with an input range of ± 500 mV through a Biosemi ActiveTwo system (Biosemi, Amsterdam, Netherlands), which replaces the conventional ground electrodes by two separate electrodes: the common mode sense (CMS) active electrode and the driven right leg (DRL) passive electrode. This common mode reference for online data acquisition and impedance measures is a feedback loop driving the average potential across the montage close to the amplifier zero. The signals were amplified and analogue band-pass filtered between 1 and 100 Hz and was digitized with 24-bit resolution.

The analysis was performed using a MATLAB toolbox described earlier^41^. Briefly, EEG spectral power density was calculated using a Welch’s method with a Hanning window function. Power was expressed as relative power for each frequency over 1–256 Hz. Average relative power in each frequency bin of each location was averaged across animals to obtain the spectrum relative to total power spectrum. For the sake of clarity in presenting this spectral data, graphs only shown the frequency range between 1 and 30 Hz and inset plots from 30 to 100 Hz.

#### Coherence

In order to describe interconnectivity between pairs of EEG electrodes, coherence measure was used, which describe level of connectivity as a value in interval [0, 1] (where 1 corresponds to complete perfect relation) for each frequency band f. Coherence is estimated as Coh(f) = |S_AB_(f)|^2^/(S_AA_(f)S_BB_(f)); where S_AB_ is the cross-spectrum between the signals A and B from two different electrodes; S_AA_, is the autospectrum of the signal A; S_BB_, is the autospectrum of the signal B. Coherence analysis allows assessment of pairwise synchronization of LFP/EEG signals to shed more light onto the interaction between different brain networks.

### Electrophysiology

#### In-vivo LTP

Rats were anesthetized with an intraperitoneal injection of urethane (ethyl carbamate, 1.5 gm/kg, i.p.), and supplemented as necessary (0.2 mL/100 g) dependent upon the response to a paw pinch. Core body temperature was monitored and maintained at 37 °C through a heating pad and rectal probe for the duration of the experiments. Once the skull was exposed, two bregma-referenced holes were drilled to insert stimulating twisted-wire bipolar and recording monopolar electrodes constructed from Teflon-coated tungsten wires (75 µm external diameter). Recordings of field excitatory post synaptic potentials (fEPSPs) were made from the stratum radiatum in the CA1 area of the right hippocampal hemisphere in response to stimulation of the ipsilateral Schaffer collateral–commissural pathway.

The electrode implantation sites were identified using stereotaxic coordinates, with the stimulating electrodes −3.4 mm posterior to bregma, −2.5 mm lateral to midline and 1.9–2.4 mm ventral, and the recording site located −4.2 mm posterior to bregma, −4 mm lateral to the midline and 2.5–3.4 mm ventral. Before the experiment, the correct placement of SC-CA1 implants were finely adjusted by altering the depth of both stimulation and recording electrodes in 10 µm increments for optimal field post synaptic potentials (fEPSP) evoking through the oscilloscope. Two epidural screws were inserted in the skull over the cerebellum served, respectively, as the reference and the recording ground. During surgery, all efforts were made to minimize animal suffering.

#### HFS for LTP induction

Stimuli were delivered using a constant current isolator unit (multichannel system MC STG4002). The induced field potential response of the SC1 was passed through the active two electrodes Biosemi amplifier (Differential amplifier, Netherlands) and digitized at 3 kHz. At the beginning of each experiment, an input–output (I/O) curve with stimulus at a frequency of 0.033 Hz and intensities ranging from 1 to 10 Volts was generated for each animal to determine the maximum fEPSP slope, and averaging five responses per intensity, then the intensity of test stimulus was set at a level that evoked an fEPSP slope of 50% of the maximum was used for all subsequent stimulations.

After the determination of I/O curves, the test stimulation was applied every 30 s before and after tetanic stimulation. For each time point measured during the experiments, five records of evoked responses at the frequency of 0.033 Hz were averaged. Baseline activity was measured every 2.5 min for at least 1 h to ensure stable baseline. The last 30 min of the baseline recording (12 time points), immediately after drug application was averaged and used as control for LTP induction. Tetanisation was induced using a high-frequency stimulation (HFS) 200-Hz protocol consisting of square pulses (0.2 msec stimulus duration, 10 bursts of 20 stimuli, 2 s inter-burst interval) at a stimulus intensity that evoked an fEPSP slope that was approximately 50% of the maximal response. fEPSP were recorded during 120 min after HFS to determine possible changes in the synaptic response of SC1 neurons. LTP measurements were derived from field EPSP ratios of the normalized slope average obtained 120-min following HFS divided by the normalized slope average collected 30 min prior to HFS. Slope of putative fEPSP were measured between the end of stimulus artefact and the trough of the negative peak. The slope of the fEPSP was calculated using a linear fit least square analysis on the 80% interval between the artefact end and the negative peak. fEPSP slopes were obtained every 2.5 min as an average of 5 responses at 0.033 Hz and were then expressed as percentage change from baseline (defined as the last 30 min prior tetanisation). LTP was defined as an increase in fEPSP slope that is maintained above 120% relative to baseline for 2 h following HFS administration. At the end of the electrophysiological study, three 30-s electrical pulses of 500 µA were delivered to produce a lesion at the end tip of the stimulation and recording electrodes and brains were harvested for histological verification of electrodes placement. Brain sections (20 µm) were examined using a light microscope. Animals with incorrect electrode placement were excluded from the study.

### Drugs

The GlyT1i (SSR504734), D-serine, and rolipram were purchased from Sigma Aldrich. SSR504734 (2.5, 10 and 40 mg/kg) was formulated in 10% CD + 1 HCl + NaCl. Rolipram (1, 3 and 10 mg/kg) was formulated in 10% CD + 1 HCl + NaCl. D-serine (20, 80 and 320 mg/kg) was dissolved in NaCl + H2O + NaOH. Drugs were administered subcutaneously in a volume of 1 ml/100 g body weight.

### Data analysis

#### EEG

Result for EEG spectrum metrics were calculated for each frequency bin and were expressed as relative total power spectra during 3 h following the administration of test drugs. Animal numbers were chosen to ensure adequate statistical power comparable to previously published papers. Data distribution was assumed to be normal and were presented as mean values with 95% confidence intervals (CI). One-way ANOVA followed by Dunnett’s post hoc test were used to assess difference in means between vehicle and different drug doses for EEG power and coherence measures while considering the heterogeneity between animals. ANOVA results are reported in figures’ captions, and mean data are visualised as box plots with significant differences based on post hoc analysis indicated by asterisks (*p-value < 0.05, **p-value < 0.01).

#### LTP

In vivo electrophysiology fEPSP responses recorded after application of drugs were expressed as percentage of change from baseline. The slope of the fEPSP was calculated using a linear fit least square analysis on the 80% interval between the artefact end and the negative peak. fEPSP slopes were obtained every 2.5 min as an average of 5 responses at 0.033 Hz and were then expressed as mean percentage change from baseline (defined as the last 30 min prior tetanisation) ± SEM. Firstly, in order to assess longitudinal changes after application of HFS, mixed-effect modelling was applied. Time after HFS was log-transformed as t_new_ = ln(1 + t_old_/5), where t_old_ is a real time expressed in minutes. This transformation allowed to linearize the data. As a next step, fEPSP relative to baseline (%) variable was modelled as t_new_ * condition + (1|animal), where condition variable is categorical variables describing vehicle and different drug concentrations. Effect of condition variable on intercept (to assess initial differences in fEPSP responses) and slop (to assess differences in attenuation of fEPSP responses in time) of the model were tested. Of primary interest there were differences between vehicle and drug. Secondly, in order to understand difference between conditions at each time point after HFS, repeated measures analysis of variance (ANOVA) followed by a post-hoc test (Dunnett’s test) were used. *p* < 0.05 was considered statistically significant.

## Results

### Effects of SSR504734, D-serine and rolipram on network oscillation and connectivity

Previous reports reveal the presence of two different theta (slow (4–6) and fast (6.5–8 Hz) and gamma bands (slow (30–50 Hz) and fast (50–100 Hz)). Both rhythms are involved in the communication between CA1 and CA3 and between CA1 and entorhinal cortex^40,42,43^ and showed sensitivity to pharmacological treatment^40^. Therefore, we evaluated LFP power in slow, and fast theta-gamma rhythms of the CA1–CA3-frontal circuit during treatment with D-serine, SSR504734 and rolipram.

**Fig. 1:**
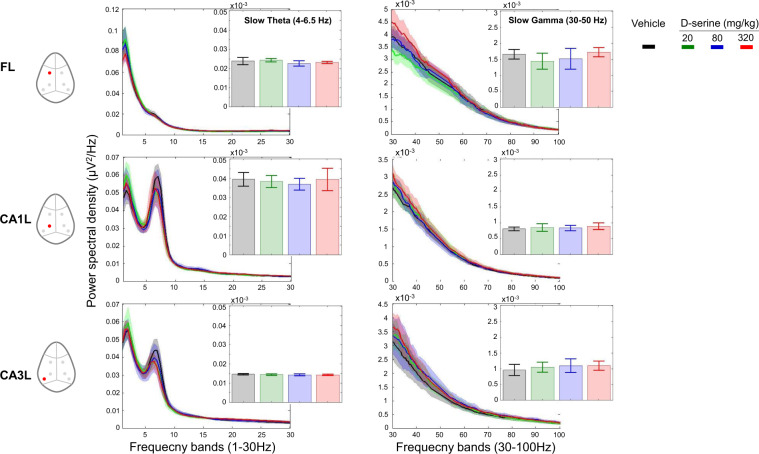
D-serine had no effect on the waking EEG power spectral density. Full power spectrum density in fronto-CA1–CA3 areas relative to total power over 1–30 Hz and 30–100 Hz for 180 min post-treatment with vehicle and D-serine (20, 80 and 320 mg/kg), *n* = 8 for each (curve panels). Inset bar panels show mean power over 4–6.5 Hz and 30–50 Hz. Note that only left hemisphere frontal, CA1 and CA3 were displayed. One-way ANOVA: (slow theta) FL: F(3, 28) = 1.15, *p* = 0.35; CA1L: F(3, 28) = 0.70, *p* = 0.57; CA3L: F(3, 28) = 0.27, *p* = 0.84; (slow gamma) F(3, 28) = 0.96, *p* = 0.42; CA1L: F(3, 28) = 0.37, *p* = 0.77; F(3, 28) = 0.54, *p* = 0.66. Post hoc analysis of EEG spectra at different frequency bands did not show significant differences between rats treated with D-serine from vehicle. Data are presented in mean (±95% CI, *n* = 8 for each condition).

No major effect was observed on slow and high theta or gamma spectra after D-serine treatment. No frequency band showed consistent changes between 0 and 100 Hz across regions from pre-injection levels and compared to vehicle (Fig. 1). In addition, there was no site-pair by frequency changes for peak coherence after the administration of different does of D-serine (Fig. 4 only mean peak slow theta coherence was displayed over the 3 h period following the administration).

**Fig. 2:**
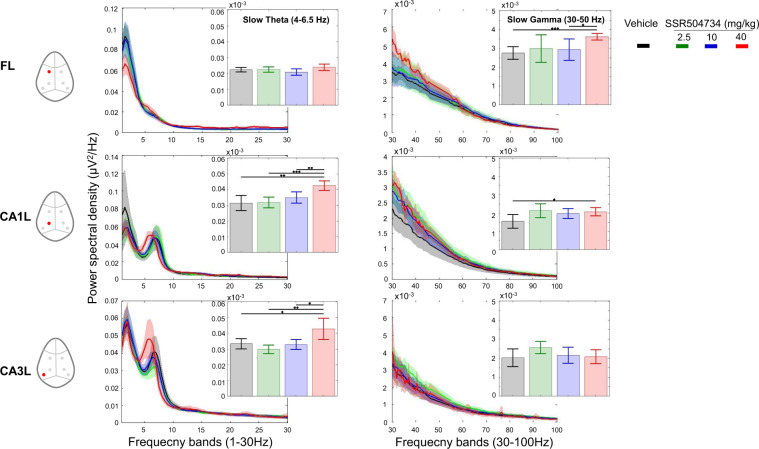
SSR504734 enhanced waking EEG power density in the slow theta oscillation at CA1–CA3 circuit as well as the slow gamma at the prefrontal cortex. Full power spectrum density in fronto-CA1–CA3 areas relative to total power over 1–30 Hz and 30–100 Hz for 180 min post-treatment with vehicle and SSR504734 (2.5, 10 and 40 mg/kg) (curve panels). Inset bar panels show mean power over 4–6.5 Hz and 30–50 Hz. One-way ANOVA: (slow theta) FL: F(3, 28) = 1,95, *p* = 0.14; CA1L: F(3, 28) = 6.3, *p* = 0.002; CA3L: F(3, 28) = 7.1, *p* = 0.001; (slow gamma) F(3, 28) = 2.92, *p* = 0.05; CA1L: F(3, 28) = 2.27, *p* = 0.10; CA3L: F(3, 28) = 0.62, *p* = 0.60. Post hoc analysis revealed that treatment increased the amplitude in the slow theta band relative to levels post administration of vehicle. Significant differences between groups based on post hoc tests are shown as an asterisk above the bars (*p < 0.05). Data are presented in mean (±95% CI, *n* = 8 for each condition).

**Fig. 3:**
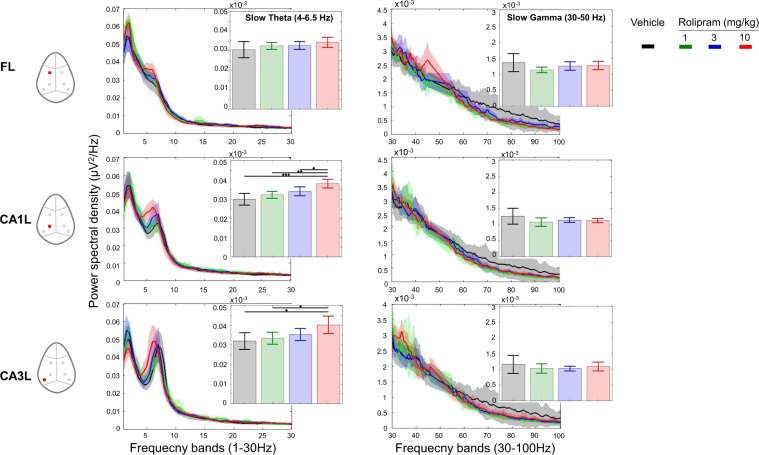
Rolipram significantly enhanced waking EEG amplitude of slow theta at CA1–CA3 circuit. Full power spectrum density in fronto-CA1–CA3 areas relative to total power over 1–30 Hz and 30–100 Hz for 180 minutes post-treatment with vehicle and rolipram (1, 3 and 10 mg/kg) (curve panels). Inset bar panels show mean power over 4–6.5 Hz and 30–50 Hz. Note that only left hemisphere frontal, CA1, CA3 and parietal cortex are displayed for animals that receive vehicle (1, 3 and 10 mg/kg). One-way ANOVA: (slow theta) FL: F(3, 28) = 1.43, *p* = 0.25; CA1L: F(3, 28) = 7.9, *p* = 0.0005; CA3L: F(3, 28) = 4.3, *p* = 0.01; (slow gamma) F(3, 28) = 1.15, *p* = 0.34; CA1L: F(3, 28) = 1.10, *p* = 0.36. CA3L: F(3, 28) = 0.4, *p* = 0.76. No major effect was observed in high spectra variables. Significant differences between groups based on post hoc analysis are shown as an asterisk above the bars (*p < 0.05). Data are presented in mean (±95% CI, *n* = 8 for each condition).

**Fig. 4:**
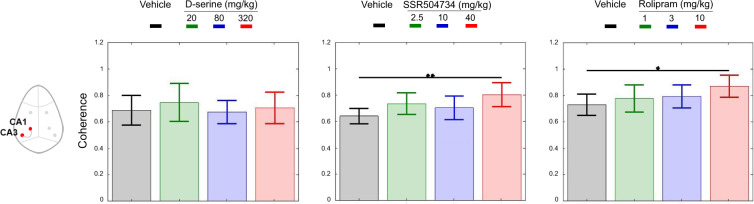
Mean peak coherence changes over slow theta oscillations 4–6.5 Hz in CA1–CA3 recording pairs at 180 min after the administration of drugs. D-serine produced no effect on the peak coherence index (F(3, 28) = 0.35, *p* = 0.78) and there was no region by frequency or site-pair by frequency coherence. In contrast, SSR504734 (F(3, 28) = 2.57, *p* = 0.07) and rolipram (F(3,28) = 1.2, *p* = 0.33) enhanced peak coherence index in the CA1–CA3 site-pairs. Note that pairs from left hemispheres were displayed for animals that received vehicle or D-serine (20, 80 and 320 mg/kg), SSR504734 (2.5, 10 and 40 mg/kg) and rolipram (1, 3 and 10 mg/kg). Significant differences between groups based on post hoc analysis are shown as an asterisk above the bars (*p < 0.05). Data are presented in mean (±95% CI, *n* = 8 for each condition).

In contrast, LFPs recorded after SSR504734 and rolipram were associated with similar pattern of power and peak coherence changes. The EEG of rats shifted to continuous synchronized activity to slow theta oscillations at the CA1 network after the administration of both drugs in the model (Figs. 2 and 3). Rolipram further synchronized slow theta at the CA3 level (Fig. 3), while no major changes were observed in the activity of other frequency bands in CA1–CA3 network. SSR504734 at the higher dose yielded additional patterns of activity consisted with enhancing effect on frontal-CA1 network gamma power (Fig. 3). Coherence of both SSR504734 and rolipram peaked at the slow theta frequency in the CA1–CA3 network (Fig. 4 middle and right panel, respectively).

**Fig. 5:**
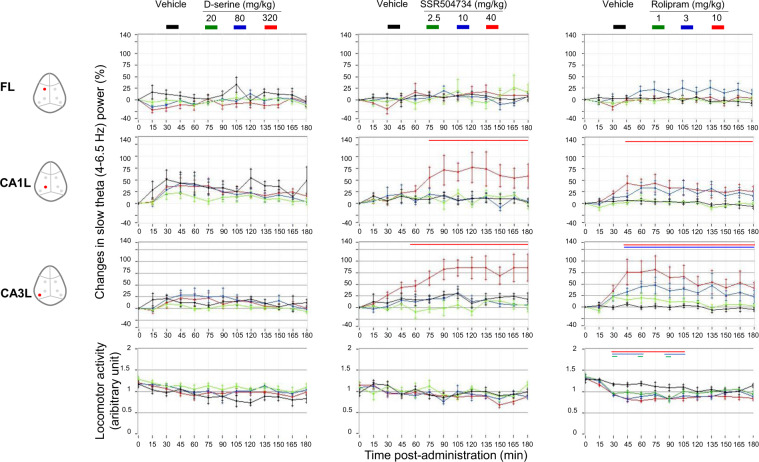
Time course changes in the EEG slow theta (4–6.5 Hz), in fronto-CA1–CA3 areas and associated activity levels during the first 3 h after the administration of D-serine, SSR504734 and rolipram. Note that treatment-induced changes in EEG activity was not associated with changes in motion levels. Values represent means ± SEM for each condition (*n* = 8). Colour coded bars above the curves indicate intervals in which oscillatory activity difference differed from vehicle.

To assess whether changes in EEG slow theta activity (4–6.5 Hz) oscillations were associated with changes in motor behaviour following different treatments, the time course of motor activity at different time points revealed a reduction in activity levels (Fig. 5).

Next, we examined whether the observed differences in hippocampal oscillation are associated with changes in synaptic plasticity.

### Effects of exogenous D-serine on LTP

**Fig. 6:**
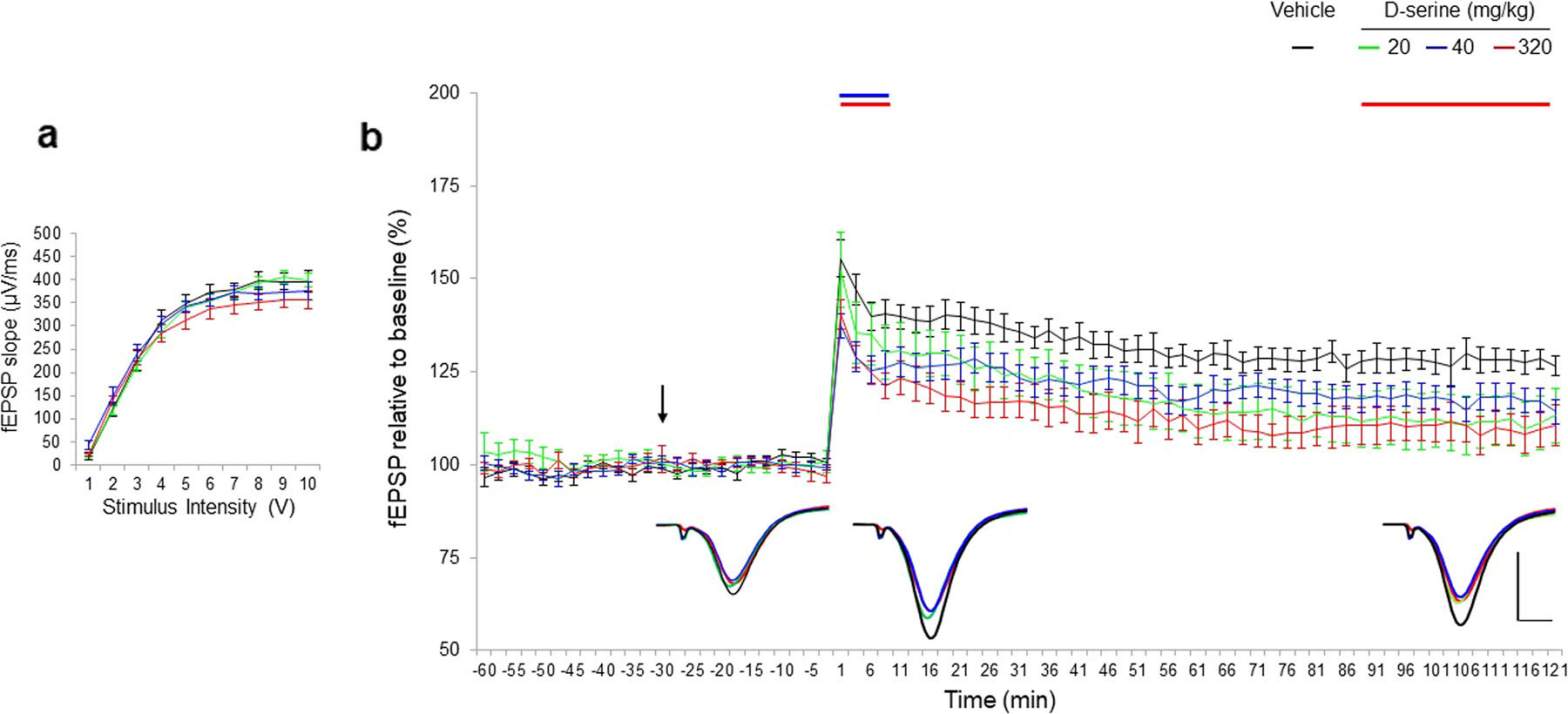
D-serine impaired LTP response to HFS at the Schaffer collateral-CA1 synapses. **a** Collective input/output curves of stimulation intensity and fEPSP slope overlap and show no significant difference in basal synaptic excitability prior treatment with D-serine. **b** Basal fEPSPs were not affected, however, the LTP response of D-serine treated subjects was significantly affected. When compared to vehicle (*n* = 9), average fEPSP slope responses showed no effect of D-serine at 20 and 40 mg/kg (green *n* = 9 and blue *n* = 9, respectively) on LTP over the 120 min periods post-HFS protocol. Average fEPSP slopes declined and show a significant reduction in STP during the first 10 min post HFS and LTP during the last recording interval particularly with the higher dose of 320 mg/kg (red *n* = 12). Values represent means ± SEM. *p* < 0.05 ANOVA. Inset represent average waveform field potentials during 30 min baseline prior tetanisation, 0–30 min and 90–120 min post-tetanisation. Horizontal bar: 1 mV, vertical bar. 5 ms. Straight lines on top of the curves indicate significance for the short-term and long-term potentiation.

The effect of exogenous D-serine (20, 80, 320 mg/kg) was investigated on LTP induction and maintenance. Baseline input/output (I/O) curves for fEPSP slopes were not significantly different confirming that the SC fibres in all dose groups had similar basal synaptic transmission (Fig. 6). D-serine had no effect on basal synaptic activity.

Mixed-effect model revealed significant effect of D-serine after the HFS tetanisation on short term potentiation “STP” (model’s intercept: *p* < 0.001, and each concentration of drug was significantly different from vehicle [*p* < 0.001]) and inhibition effect on LTP in time (model’s slope: *p* < 0.001, and each concentration of drug, except for 40 mg/kg, was significantly different from vehicle [*p* < 0.001]). Absence of significant difference of the 40 mg/kg injection on model’s slope (*p* = 0.77) characterizes, that LTP after vehicle and 40 mg/kg injections were inhibited at the same rate.

More precisely, after HFS tetanisation, D-serine impaired STP (−7.2%, −16.1% and −17%: *p* = 0.02, respectively) and the expression to LTP (over 2 h). Further analysis into the last period of the recording session revealed a significant inhibitory effect on LTP maintenance, particularly with the higher dose (90–120 min post-HFS, −18%: *p* = 0.03) (Fig. 6).

### Effects of SSR504734 on LTP

**Fig. 7:**
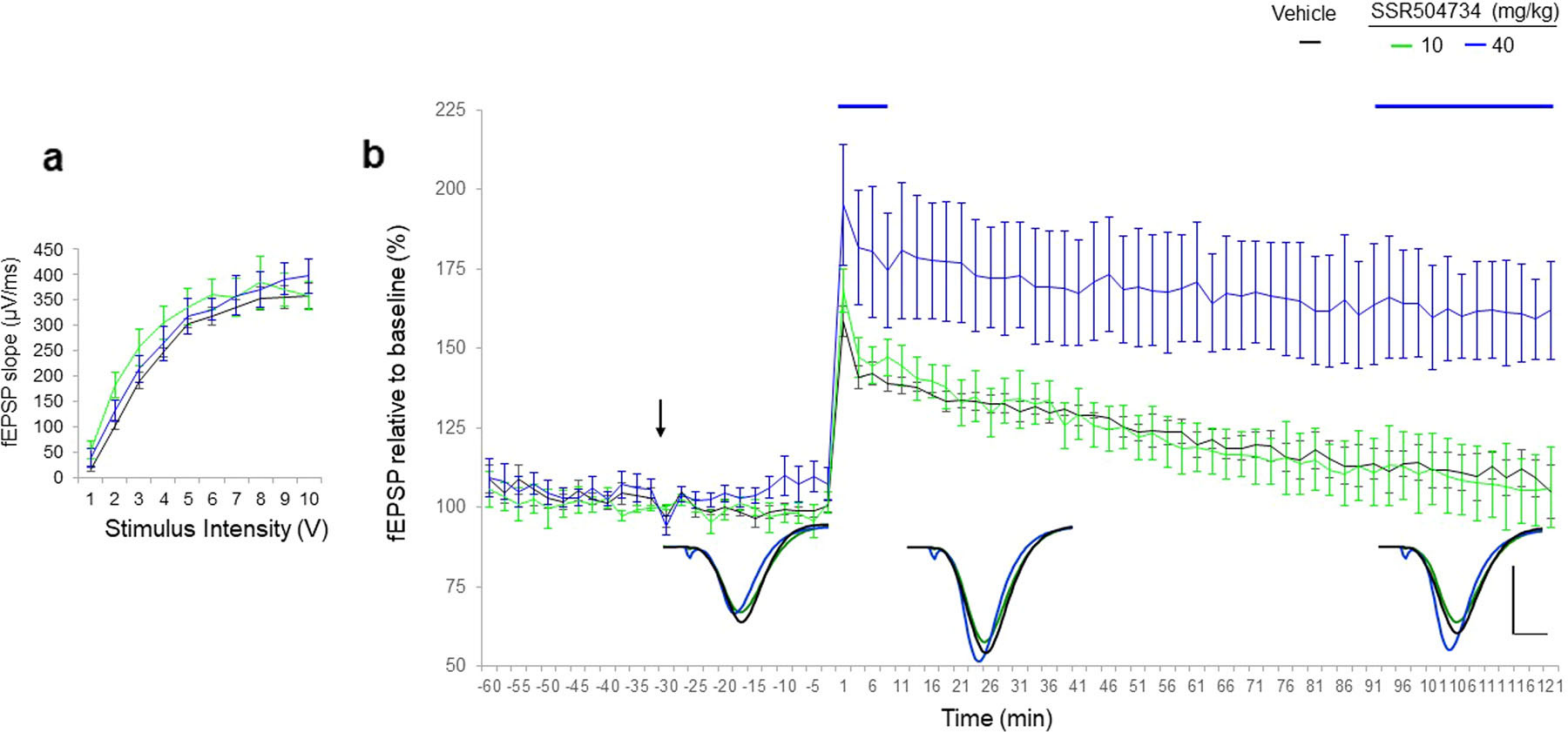
SSR504734 enhanced LTP response to HFS at the Schaffer collateral-CA1 synapses. **a** Collective I/O curves of stimulation intensity and fEPSP slope overlap and show no significant difference in basal synaptic excitability prior treatment with SSR504734. **b** Average fEPSP slope responses show that SSR504734 at the dose of 10 mg/kg, had no effect on the induction of LTP (green, *n* = 5) as compared to vehicle (black, *n* = 5). At the dose of 40 mg/kg (blue, *n* = 6), SSR504734 enhanced LTP as compared to vehicle. Values represent means ± SEM. Values represent means ± SEM. *p* < 0.05 ANOVA. Inset represent average waveform field potentials during 30 min baseline prior tetanisation, 0–30 min and 90–120 min post-tetanisation. Horizontal bar: 1 mV, vertical bar. 5 ms. Straight lines on top of the curves indicate significance for the short-term and long-term potentiation.

The effect of increased glycine levels via reuptake inhibition on LTP expression was investigated. I/O curves were not significantly different confirming that the SC fibres in all dose groups were of a similar excitability (Fig. 7). SSR504734 had no influence on basal synaptic transmission.

Mixed-effect model did not reveal significant effect of SSR504734 after the HFS tetanisation on STP (model’s intercept: *p* = 0.058), but the effect on enhancement of LTP in time was significant (model’s slope: *p* < 0.001).

More precisely, SSR504734 at 10 mg/kg failed to enhance the slope of fEPSPs above vehicle levels during the entire recording session of 2 h post-treatment. However, there was a significant effect of SSR504734 at the dose of 40 mg/kg on LTP maintenance as revealed during the last period of the recording session after HFS (90–120 min post-HFS, +41%: *p* = 0.001).

### Effects of rolipram on LTP response

**Fig. 8:**
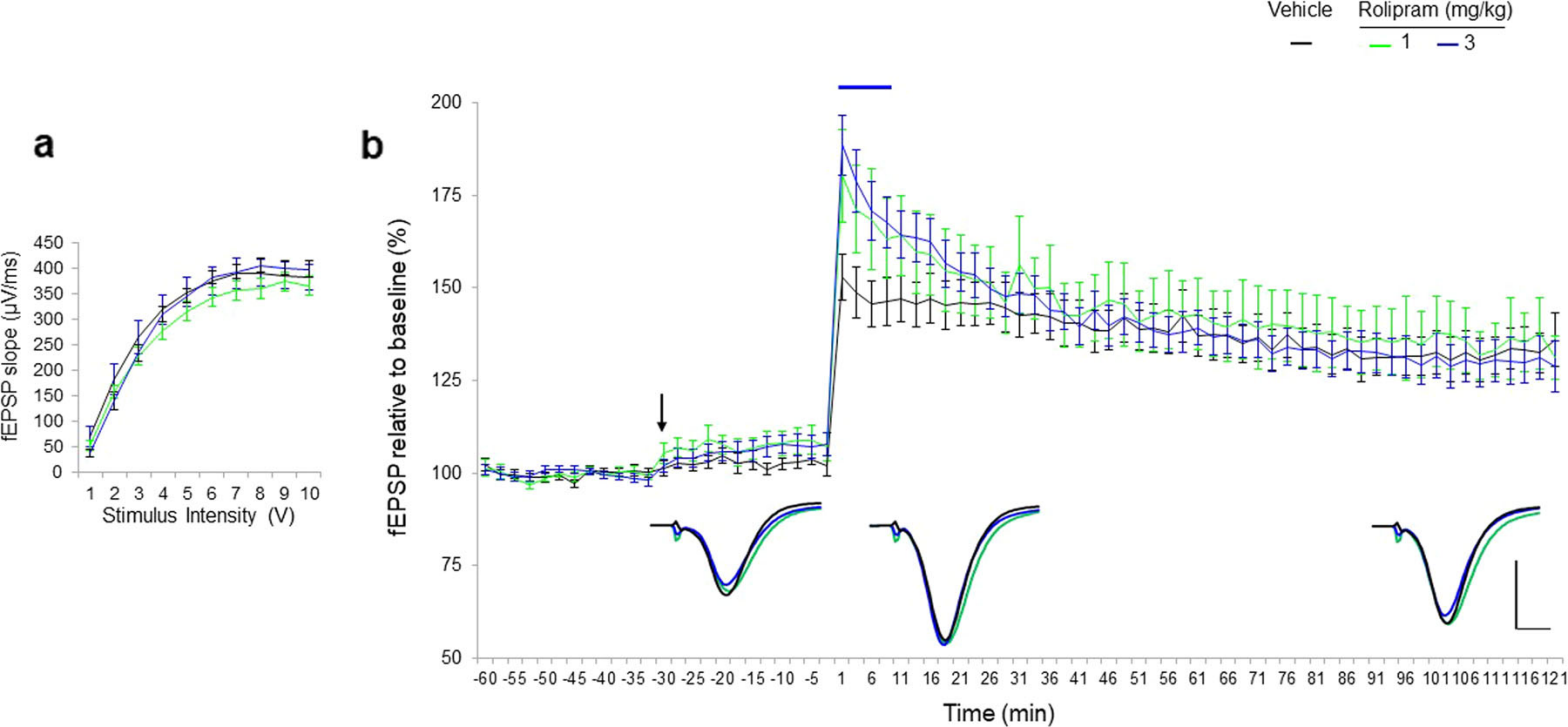
Rolipram enhanced short term plasticity response to HFS at the Schaffer collateral-CA1 synapses. **a** Collective I/O curves between stimulation intensity and fEPSP slope show no significant difference in fibre sensitivity between groups. Rolipram leads to short lived alterations in excitability or in ability to express synaptic potentiation in the hippocampi CA1 area by facilitating STP, which did not express into LTP. **b** Average fEPSP slope responses showed that rolipram at the dose of 1 mg/kg (*n* = 11) had no effect on STP and LTP variables, however rolipram at the dose 3 mg/kg (red, *n* = 11) increased STP as compared to vehicle (black, *n* = 8). Values represent means ± SEM. Values represent means ± SEM. *p* < 0.05 ANOVA. Inset represent average waveform field potentials during 30 min baseline prior tetanisation, 0–30 min and 90–120 min post-tetanisation. Horizontal bar: 1 mV, vertical bar. 5 ms. Straight lines on top of the curves indicate significance for the short-term and long-term potentiation.

The effect of increasing cAMP levels with the selective blocker of PDE4 reuptake inhibition on LTP expression was studied. The results showed no significant difference between group I/O curves confirming there was no difference in the excitability SC-CA1 synapses in the study groups (Fig. 8). Similarly, basal synaptic transmission was not affected by application of rolipram.

Mixed-effect model revealed significant effect of rolipram after the HFS tetanisation on STP (model’s intercept: *p* < 0.001) and enhancement effect on LTP in time (model’s slope: *p* < 0.001).

Detailed analysis of difference driving above’s significance results showed, that the dose of 3 mg/kg, rolipram led to the increased STP (+25%, *p* = 0.04, Fig. 8), however, this transient potentiation did not express into LTP. At the lowest dose of 1 mg/kg, rolipram had no significant effect on STP and the expression into LTP as compared to vehicle (Fig. 8).

## Discussion

In the present study, we sought to define patterns of network oscillatory activities and plasticity amongst anatomically and functionally connected brain region after modulation of both NMDA and cAMP signalling. Both SSR504734 and rolipram enhanced network slow theta oscillations, connectivity in the CA1–CA3 circuit. SSR504734 enhanced LTP response, whereas the plasticity was short-lived to STP with rolipram. Unlike glycine, increased levels of D-serine had no effect on network oscillations and limits the induction and expression of LTP.

Neural oscillations are critical mechanisms allowing dynamic coupling of intra- and inter-regional brain regions during information processing^44,45^. For instance, theta oscillatory rhythm derived from concerted generators including cholinergic and GABAergic, as well as glutamatergic networks in different locations, plays a key role in the function of the hippocampus and associated cortical networks^46^. Afferent cholinergic and glutamatergic input from the medial septum-diagonal band of Broca, as well as from CA3 and entorhinal cortex provide support for theta oscillations at the CA1 circuit^46^. Modulation of the glutamatergic NMDA signalling through local modulation of septal circuit mediates the generation of hippocampal theta rhythm^47^. Theta oscillations may facilitate synchrony between hippocampus and prefrontal cortex network required for learning and memory consolidation^48^.

Gamma oscillations (30–100 Hz) are prominent in the cortical-hippocampal network and have been shown to appear during a variety of memory tasks in rats, monkeys, and humans^43,49^. Gamma rhythms occur as two distinct variants that are thought to route different streams of information entering hippocampal subfield CA1^42,50^. Slow gamma (30–50 Hz) may promote direct inputs from CA3 to CA1, which is believed to be associated with memory retrieval^51,52^. Fast gamma (50–100 Hz) may facilitate direct transmission from the medial entorhinal cortex that transmit ongoing spatial information^51,53^.

Functional roles of theta and gamma oscillations in mnemonic processes has been established^54^, and while disruption of this oscillatory rhythms has been associated with cognitive deficits described in psychiatric and neurodegenerative disorders^55,56^. Pharmacological and several new therapeutic techniques, such as transcranial magnetic stimulation, transcranial direct current stimulation, and closed-loop stimulation, have profound direct and indirect effects on ongoing oscillatory activity in the brain^57^, and restoration of theta and gamma like rhythmicity restores learning and memory capabilities in rats^58–60^.

### SSR504734 and rolipram but not D-serine enhanced network oscillations and connectivity

Abundant evidence points to the importance of NMDA receptors in patterning neuronal networks and synaptic transmission. Positive modulation of the co-agonist binding site on the NMDA receptor has been proposed as a novel therapeutic approach to overcome negative symptomatology and cognitive dysfunction seen in those diseases^61^. Glycine and D-serine are endogenous ligands to the NMDA modulatory site^62^, and ligands modulating NMDA receptor transmission, have being developed such as GlyT-1 inhibitors to potentially elevate brain glycine or targeting enzymes, such as d-amino acid oxidase (DAAO) to slow the breakdown and increase the brain level of D-serine^63,64^. In the present study we further evaluated effects of the glycine inhibitor, exogenous D-serine or modulating cAMP levels on synaptic network oscillations and plasticity. The oscillatory activity across the CA1 network of structures studied in the present work was dominated by changes in the slow theta rhythm and related coherent activity. Thus, indirect modulation of NMDA signalling through inhibition of the GYT1 may support the hypothesis that the release of endogenous D-serine from astrocytes did not saturate and, therefore, enough to generate and entrain synchronized theta rhythm in CA1–CA3 network during information processing. However, exogenous supply of D-serine failed to promote similar changes in network oscillation and connectivity, likely because D-serine levels might have reached saturation and therefore shunt down synchronized theta rhythm. In addition, SSR504734 enhanced slow gamma at the frontal and CA1 structures, which of rhythm has been hypothesized to promote memory retrieval.

Therefore, the evoked slow theta and gamma network oscillations at the CA1 and frontal networks, respectively suggests a positive modulation effect of the glycine site of the NMDA receptor on attentional processing and memory operations phases.

Alterations in cAMP signalling are thought to contribute to neurocognitive and neuropsychiatric disorders. Phosphodiesterases play an essential role in orchestrating the compartmentalized degradation of cAMP, leading to local changes in cAMP signalling in specific subcellular domains in the cell^65^. The cAMP signalling pathway is a second messenger that has a key role in several intracellular cascades, including the cAMP/protein kinase A (PKA)/cAMP response element-binding protein (CREB) pathway which is critically involved in learning and memory^5^. Changes in cAMP levels has been shown to regulate theta activity, and rolipram administered intravenously evoked an arousal EEG pattern period (low amplitude fast waves) in the cortical EEG and synchronization of the hippocampal theta waves with increased voltages^66^. In the amygdalo-hippocampal pathways, an increase in intracellular cAMP concentration facilitates regular firing and oscillatory activity at the theta frequency range^67^, supporting synaptic signal transfer between those functionally connected neuronal populations during retrieval of conditioned fear. Disruption of theta activity results in spatial memory deficits, whereas the restoration of theta-like rhythmicity reverse learning deficits in rats^59,60^. In the present work, the enhanced slow theta activity confirmed the potential positive modulatory effect of rolipram on neural networks.

### Glycine transporter inhibitor and rolipram, but not D-serine, enhanced in vivo plasticity

#### LTP and glycine

Activity-dependent synaptic plasticity, such as NMDAR-dependent LTP has been proposed as a cellular mechanism underlying learning and memory in the brain^68,69^. The GlyT1 antagonist NFPS increased NMDAR channel opening in a dose-dependent manner in Sprague-Dawley prefrontal cortex slices^70^. Similarly, the antagonist CP-802,079 enhanced LTP induced by HFS in hippocampal slices^14^. However, the effects of both these antagonists appear irreversible. Depoortère et al.^71^ first reported on the neurochemical, electrophysiological and pharmacological characteristics of the selective, reversible GlyT1 inhibitor SSR504734. The compound enhanced in a concentration-dependent manner the NMDA component of CA1 excitatory postsynaptic currents therefore further confirming the role of the GlyT1 in NMDA excitability. In the present study, SSR504734 enhanced the LTP expression at the highest dose of 40 mg/kg for the entire 2-h post-tetanisation recording period. The significantly enhanced and enduring LTP is likely the result of increased NMDAR channel openings^70^ as this would cause a larger postsynaptic influx of Ca^2+^ and activation of LTP maintenance mechanisms.

The lower dose of SSR504734 given, 10 mg/kg had no effect on LTP as compared to the vehicle. Microdialysis studies in the prefrontal cortex and nucleus accumbens revealed a significant increase in the extracellular glycine concentrations for 45–180 min after the application of SSR504734 at the dose of 10 mg/kg^71,72^. According to this time frame, the HFS in this study fell 15 min before synaptic glycine concentrations were increased. It is, therefore, possible that SSR504734 (10 mg/kg) successfully increased glycine concentrations in this study. However, as this would not have occurred prior to or during the tetanisation, the extra glycine at the synapse following GlyT1 inhibition would not have been able to enhance NMDAR excitability before administration of the HFS. As a result, the NMDAR-dependent LTP of CA1 would not have been enhanced by the GlyT1 inhibition. The 40 mg/kg dose may also have a faster time frame for glycine concentration increase following injection due to the higher concentration of compound available. Extracellular increase in glycine concentration has not been investigated at doses above 10 mg/kg of SSR504734^71,72^. A subsequent microdialysis study could confirm the hypothesis that HFS was administered before peak glycine levels with the 10 mg/kg dose and that this effect was shifted rightwards following 40 mg/kg.

SSR504734 has also been investigated in mice performing in an operant delayed alternation cognitive task^63^. Success in the task, which relies on working memory, became more difficult with longer time intervals and delays above 8 s between trials proved to be challenging. Animals treated with the dose of 30 mg/kg successfully completed tasks with intervals of 12–18 s, intervals at which control animals could no longer perform above chance levels. At intervals of 12 s, SSR504734 (30 mg/kg) enhanced the percentage of correct choices as did 10 mg/kg, however, this was not a significant effect. Other studies that used SSR504734 showed efficacy in behavioural studies with doses higher than 10 mg/kg^73,74^. Therefore, the present results support the earlier behavioural studies. SSR504734 1(0 mg/kg) may enhance glycine levels but not sufficient to overcome the effect of other factors in an intact brain. Indeed, the other NMDAR coagonist D-serine exhibits higher affinity for the strychnine-insensitive binding site^9^. This binding competition could prevent intermediately increased glycine levels from having a detectable effect on LTP or behavioural response. The results of this work suggest that reuptake inhibition with SSR504734 and the two GlyT1 inhibitors NFPS^12^ and CP 802,079^14^ did indeed lead to increased synaptic levels of glycine, therefore increasing its role as a facilitator of NMDAR excitability and the enhancement of LTP following HFS. The results suggest a beneficial effect of GlyT1 inhibitors on hippocampal-dependent forms of memory deficient, and further provide a compelling rationale for using GlyT1 inhibitors to indirectly potentiate NMDA receptor functions.

#### LTP and D-serine

Endogenous D-serine can be released in an activity-dependent manner and, in turn, contributes to the induction of LTP and LTD^19,75^. D-serine is a more potent agonist of the NMDAR than glycine^9^, and D-serine is moderately better than glycine in penetrating the blood–brain barrier when administered systemically, therefore, it was expected that the high doses of D-serine given in this experiment would potentiate the LTP response. However, this was not the case with the middle dose (40 mg/kg) had a slightly depotentiation effect on LTP levels and the highest dose (320 mg/kg) decreasing LTP response. Whilst some groups have reported enhancing LTP with D-serine application^16,76^, the consensus in the literature is that exogenous D-serine has no effect on LTP in wild-type animals even though its increased NMDAR-mediated post-synaptic responses in hippocampal slices^77–79^. D-serine treatment alone has no effect on LTP induction, while it decreased the basal glutamatergic neurotransmission, which weakens the efficacy of the agonist on synaptic plasticity and depresses basal AMPA receptor-mediated neurotransmission in young animals. This property may, therefore, limit the potency of the agonist to increase the magnitude of synaptic plasticity, especially in aged rats as AMPAR-mediated neurotransmission was reduced in these animals^80,81^. D-serine had no effect on spatial learning and memory per se^82^, whereas exogenous application of D-serine has however been shown to restore potentiation in aged animal models of decreased D-serine concentrations^77^ or in pharmacologically induced glial metabolism disruption^79^. Another difference that may explain discrepancies of results may be related to stimulation protocols used to induce LTP. In both groups that showed an enhancement of LTP under control conditions have used theta-burst protocol, which may activate different signalling pathways as compared to HFS as well as induce a different pattern of Ca^2+^ signalling^83^. However, plasticity studies that used HFS protocols did not reveal significant increase of the LTP response following D-serine, which may cause endogenous D-serine levels to saturate following tetanisation and shunts inhibition of afferent inputs which thus display a depression (an LTD-like effect) instead of an LTP at the soma^78^. This may explain a negative trend in basal synaptic activity and the LTP responses that were seen in this study using young, wild type rats and HFS protocol.

The novel finding reported here is that the highest dose of exogenous D-serine reduced synaptic potentiation below control levels, which could lead to receptor internalization. Both D-serine and glycine binding have been suggested to prime the NMDA receptor for clatherin-dependent endocytosis upon glutamate binding and receptor activation in hippocampal cells^84^. In this case, the high dose of D-serine would increase the number of receptors primed for internalisation. Upon application of the HFS, which would cause a large glutamate release, postsynaptic depolarisation would cause NMDA receptor endocytosis. If an enough NMDA receptor were internalised this would reduce the levels of signalling Ca^2+^ and therefore reduce the activation of LTP signalling mechanisms. As the study by Nong et al.^84^ focussed more prominently on the role of glycine in NMDA receptor internalisation, further immunocytochemical assays using D-serine could strengthen this hypothesis.

Another possibility for the decreased LTP response after high dose of D-serine may result in synaptic excitotoxicity. Indeed, high concentrations of this amino acid have been measured in pathological, neurotoxic states such as cerebral ischemia^85^. In AD, amyloid-β has been shown to induce D-serine release from microglia leading to neurodegeneration^86^. It has also been reported that exogenous D-serine has a dose-dependent bell-shaped effect on LTD magnitude^75^, which could reflect changes in the LTD/LTP threshold as set out in the Bienenstock, Cooper, and Munro (BCM) model. Accordingly, the high level of D-serine 320 mg/kg may be closer to the LTP threshold than lower or higher doses of exogenous D-serine and inducing a lower level of LTP than under vehicle or 320 mg/kg conditions. However, to our knowledge it has not been investigated whether this is also the case for LTP as single concentrations of D-serine were generally used in previous studies.

Whilst both glycine and D-serine directly modulate the excitability of NMDA receptors and enhance NMDAR-mediated synaptic transmission, clinical trials involving direct administration of both amino acids produced mixed results in improving cognitive impairments in schizophrenic patients^87^. The present study provides evidence that D-serine controls NMDAR-dependent LTP, whilst glycine influence neurotransmission at a different level, by activating extrasynaptic glycine receptors distributed along the apical dendrite. Future studies will evaluate mechanistic approaches targeting D-serine modulatory sites, for example by inhibition of the enzyme d-amino acid oxidase (DAAO), which slows the break-down of D-serine, or by its transporter, the alanine-serine-cysteine-1 (Asc-1).

#### LTP and rolipram

Alterations in the activity of PDE4 has been associated with cellular mechanisms underlying structural and synaptic damage in experimental models of mood and neurological diseases^6^. Pharmacological inhibition of PDE4 activity promotes synaptic plasticity and memory^88^. Mice lacking all PDE4D isoforms display either memory enhancements or impairments, depending on the task used^17^. The PDE4 selective inhibitor, rolipram, prevents memory deficits associated with sleep loss^89^, aging^90^, muscarinic or NMDA receptor blockade^39^, and mouse models of Alzheimer’s disease^91^. LTP can be induced in the SC-CA1 synapse of the hippocampus by stimulation in the theta frequency range (5–12 Hz), an effect that depends on activation of the cAMP pathway^92^. The amyloid beta-induced inhibition of LTP in slices was reversed following direct application of rolipram^93^.

In this study, an enhancement of early phase of LTP was observed following the administration of rolipram at 3 mg/kg. This would appear contradictory with the hypothesised role of the cAMP/PKA pathway in late memory consolidation and late-LTP^36^. Late-LTP is a more persistent and robust form of LTP lasting for 8–10 h that requires PKA activation for protein synthesis and to facilitate LTP maintenance^94^. Late-LTP can be induced using a strong tetanisation procedure such as the repetition of HFS trains^94^. Similar late-LTP levels have been recorded in vitro and in vivo using application of rolipram to enhance cAMP levels^39,88^. Wiescholleck and Manahan-Vaughan^39^ reported that this dose in vivo caused a transient chemically induced potentiation lasting an hour without tetanisation. However similar doses of rolipram in vitro show an effect on basal synaptic transmission and have been reported to have a negative impact on LTP perhaps through a toxic effect^88^. The amplification of transient cAMP by rolipram and its impact on LTP levels is sensitive to the time at which the HFS protocol is administered^88^. The enhancing effect of rolipram was only seen when the hippocampal slice was perfused with the compound during tetanisation, however, LTP was no different from saline if the perfusion occurred after HFS^88^. Most likely the highest dose used in this study transiently amplified the cAMP to elicit a potentiation but not sufficiently to fully activate CREB signalling or AMPA insertion at the synapse^95^. The enhancement of LTP by rolipram, although transient, shows that the HFS model is responsive to enhanced cAMP levels following the inhibition of PDE4. The cAMP/PKA path is not the only signalling pathway regulated by the PDEs; cGMP and the related PKG also activate the transcription factor CREB, which may be involved in earlier LTP and memory consolidation to the cAMP pathway^36^. Therefore, a combined enhancement of both cAMP and cGMP could activate mechanisms whilst also inducing the protein processes necessary for facilitating late-LTP.

**Fig. 9:**
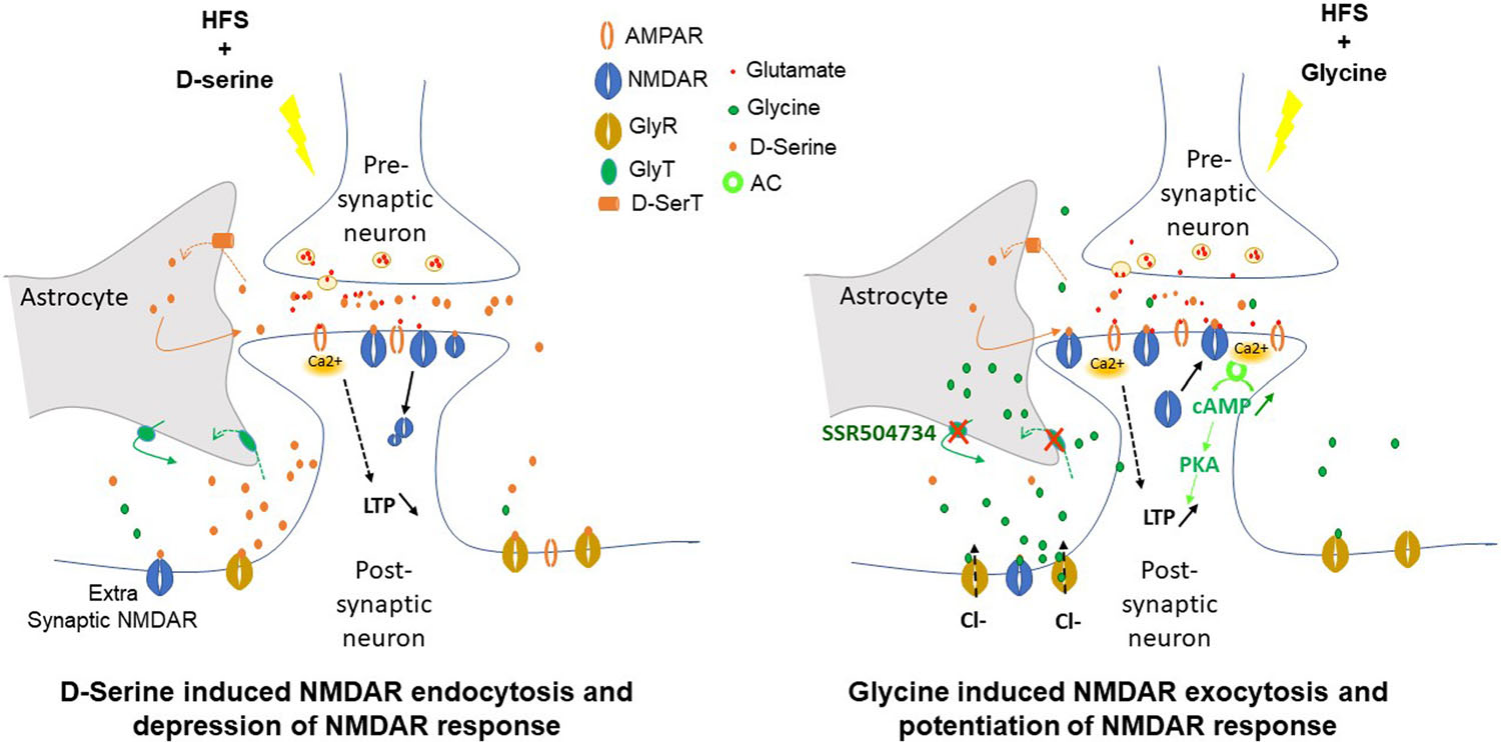
Schematic depicting the relationship between changes in the levels of D-serine and glycine with synaptic response at the level of NMDA receptors. Glutamate can be released as a gliotransmitters by either Ca^2+^-dependent vesicular release or efflux from transporters or large-conductance ion channels. Presynaptic glutamate release triggers postsynaptic activation through the binding to membrane NMDA receptors. In the synaptic cleft, glutamate can be taken up by transporters located in astrocytes and converted to glutamine and GABA. Glutamate binds to AMPA receptors on astrocytes, stimulates the release of D-serine, which is synthesized from L-serine via serine racemase in astrocytes. D-serine can be released via either vesicular or non-vesicular mechanisms and activate neuronal NMDA receptors. Under HFS, the increased glutamatergic levels in the synaptic cleft and binding of released D-serine to glycine modulatory sites on the NMDA receptors lead to overactivation of postsynaptic neurons through upregulated NMDA and mGluR receptors, which may result in endocytosis of NMDA receptors and depression of NMDAR signalling and LTP response. GlyT-1 inhibitors increase extracellular glycine levels by inhibiting GlyT-1 in glial cells, and increased glycine levels elicits NMDAR exocytosis, and stimulation of glycine modulatory sites on the NMDA receptors to potentiate NMDA receptor signalling and LTP response.

The present study provides evidence that the glycine modulatory site was required for the induction of NMDAR-dependent LTP and connectivity whilst exogenous D-serine negatively influenced neurotransmission. Unlike glycine, increased level of D-serine does limit the induction and expression of LTP in the rat CA1. We hypothesized that high levels of D-serine might results in a shunt of synaptic inputs to downregulate NMDARs currents and eventually leading to synaptic depression after application of HFS at the somatic level (Fig. 9). Our observations that rolipram elicited a transient induction of short-term form of potentiation that was not facilitated into late phase of LTP, raises the likely possibility that transiently increased cAMP levels immediately before tetanisation attenuated the molecular machinery involved in mediating the late phase of LTP in the SC-CA1 synapses.
